# Research on the effects of desflurane and sevoflurane on proliferation and migration of breast cancer cells

**DOI:** 10.1038/s41598-026-52017-5

**Published:** 2026-05-09

**Authors:** Oğuz Kaan Şimşek, Aliye Çoruh, Ahsen Güler, Ayşe Doğa Karipçin, Zühal Hamurcu

**Affiliations:** 1 Department of Anesthesiology and Reanimation, Faculty of Medicine, Erciyes University, Melikgazi, 38039 Kayseri, Turkey; 2 Department of Medical Biology, Faculty of Medicine, Erciyes University, Kayseri, Turkey; 3 Betül-Ziya Eren Genome and Stem Cell Center, Erciyes University, Kayseri, Turkey; 4 Gevher Nesibe Genome and Stem Cell Institute, Erciyes University, Kayseri, 38039 Turkey

**Keywords:** Sevoflurane, Desflurane, Proliferation, Apoptosis, Migration, In vitro, MDA-MB-231, MCF-7, MCF-10A, Cancer, Cell biology, Drug discovery, Oncology

## Abstract

This study aimed to evaluate the biological effects of sevoflurane and desflurane on breast cancer cell lines representing different molecular subtypes under in vitro conditions. Specifically, the effects of these inhalational anesthetic agents on parameters associated with cancer progression, including cell proliferation, colony formation, apoptosis, and migration, were comparatively investigated. Triple-negative breast cancer cells (MDA-MB-231), estrogen receptor-positive cells (MCF-7), and non-tumorigenic breast epithelial cells (MCF-10 A) were used. Cells were exposed to clinically relevant concentrations of sevoflurane (3.6%) and desflurane (10.3%) for 3 h, followed by 24 h of incubation. Cell viability (MTS assay), colony formation, wound healing (migration), Annexin V apoptosis analysis, and Western blot analysis of apoptosis-related proteins were performed. Exposure to desflurane and sevoflurane significantly reduced the viability of MDA-MB-231 and MCF-7 cells (*p* < 0.0001), whereas no significant change was observed in MCF-10 A cells. Colony formation was significantly suppressed only in MDA-MB-231 cells, with a more pronounced effect observed following desflurane exposure. Neither agent significantly affected cell migration. Apoptosis rates significantly increased in MDA-MB-231 cells treated with desflurane (*p* < 0.0001). Western blot analysis demonstrated that desflurane increased p53 expression in MDA-MB-231 cells, whereas sevoflurane reduced its expression; no significant alterations were observed in PARP-1 and AIF levels. These findings suggest that desflurane exerts proliferation-inhibitory and apoptosis-inducing effects, particularly in triple-negative breast cancer cells. Sevoflurane demonstrated comparatively weaker effects. Overall, our results indicate that volatile anesthetics may directly influence cancer cell biology; however, their clinical implications require further validation in in vivo and clinical studies.

**Supplementary Information:**

The online version contains supplementary material available at 10.1038/s41598-026-52017-5.

## Introduction

Breast cancer remains one of the leading causes of cancer-related mortality among women worldwide. Surgical resection continues to represent the cornerstone of curative treatment for this disease^1^. Emerging clinical evidence suggests that anesthetic agents and techniques used during breast cancer surgery may influence tumor recurrence and metastatic progression^2^.

Volatile inhalational anesthetics, including sevoflurane, desflurane, and isoflurane, are widely used in routine clinical practice. However, data regarding their direct biological effects on cancer cells remain limited and, in some cases, conflicting. Previous in vitro investigations have suggested that the choice of anesthetic agent may influence cancer cell behavior and potentially affect recurrence and metastatic potential^3^. Conversely, other studies have demonstrated variable effects depending on tumor type and experimental conditions^4–6^.

Experimental studies have reported an association between desflurane exposure and enhanced metastatic potential, as well as poorer prognosis in colorectal cancer models^5^. However, retrospective clinical studies evaluating anesthetic technique and cancer recurrence have yielded inconsistent findings^6^. While experimental studies primarily provide insight into cancer cell behavior under controlled conditions, clinical studies reflect multifactorial patient outcomes influenced by numerous variables. Therefore, whether specific anesthetic regimens influence long-term oncological outcomes remains an area of ongoing investigation, particularly regarding their long-term clinical relevance.

Therefore, the present study aimed to systematically investigate the effects of desflurane and sevoflurane, two commonly used volatile anesthetics, on proliferation, colony formation, migration, and apoptosis in triple-negative breast cancer (MDA-MB-231), estrogen receptor-positive breast cancer (MCF-7), and non-tumorigenic breast epithelial cells (MCF-10 A) cells under in vitro conditions.

## Materials and methods

### Cell lines, culture conditions, and reagents

Human TNBC cells (MDA-MB-231) and estrogen receptor positive (ER+) breast cancer cells (MCF-7) and non-tumorigenic breast epithelial cells (MCF-10 A) were obtained from the American Type Culture Collection (ATCC, Manassas, VA, USA). Breast cancer cell lines were cultured in Dulbecco’s Modified Eagle’s Medium/Nutrient Mixture F-12 (DMEM/F-12) supplemented with 10% fetal bovine serum (FBS; Sigma-Aldrich, St. Louis, MO, USA). MCF10 A cells were cultured in DMEM/F12 supplemented with 5% horse serum, epidermal growth factor, hydrocortisone, insulin, and cholera toxin (Calbiochem). Cells were cultured at 37 °C in a humidified incubator with 5% CO_2_.

### Cell culture and volatile agent application

Cells were seeded at densities appropriate for each experimental assay and allowed to adhere for 48 h under standard culture conditions (37 °C, 5% CO₂).

Subsequently, the cells were placed in a specially designed 1.5 L, airtight gas chamber (Billups-Rothenberg MIC-101) equipped with inlet and outlet valves. The cells were then exposed to a gas mixture containing 21% O_2_ and 5% CO_2_ along with either 3.6% sevoflurane or 10.3% desflurane for a duration of 3 h at 37 °C. These concentrations correspond to approximately 1.7 minimum alveolar concentration (MAC) equivalents in humans and were selected to reflect clinically relevant exposure levels^7,8^. Following exposure, the cells were returned to standard culture conditions and incubated for 24 h. Control cells were exposed to the same gas mixture (21% O_2_ and 5% CO_2_) under identical conditions, without volatile anesthetic exposure. In all groups, the culture was terminated, and subsequent analyses were performed at the end of the 24-hour incubation period.

### Cell viability and proliferation assays

Cell viability and proliferation were assessed using the MTS assay (Promega, Madison, WI, USA) as previously described in our earlier study^9^. A mixture of MTS reagent and phenazine methosulfate (PMS) at a 20:1 (v/v) ratio was added to each well. For the cell proliferation and viability assay (MTS), MDA-MB-231, MCF-7, and MCF10 A cells were seeded into 96-well plates at a density of 1.20 × 10^3^ cells/well. At 48 h post-seeding, cells were exposed to the gas mixtures containing 3.6% sevoflurane or 10.3% desflurane for 3 h. Control groups were simultaneously exposed to 21% O_2_ and 5% CO_2_ under the same conditions, without the volatile anesthetic application.

Following gas exposure, the cells were incubated for an additional 24 h under standard culture conditions. A solution containing MTS and phenazine methosulfate (PMS) (20:1 v/v) was then added to the cells. After 3 h of incubation at 37 °C, the number of viable growing cells was estimated by measuring the absorbance at 490 nm using an ELISA reader, based on the generation of formazan by the metabolically active cells^10^.

### Colony formation assays

To investigate the effects of sevoflurane and desflurane on the proliferation and colony formation capacity of breast cancer and non-tumorigenic breast epithelial cells, The clonogenic assay was performed as previously described in our study protocol^11^, with minor modifications. Cells were seeded into 6-well plates at a density of 1.5 × 10^3^ cells/well. They were subsequently treated for 3 h with gas mixtures containing either 3.6% sevoflurane or 10.3% desflurane, along with 21% O_2_ and 5% CO_2_. The control group was simultaneously subjected to the same conditions, receiving only 21% O_2_ and 5% CO_2_ without the volatile anesthetic.

Following gas exposure, cells continued to be cultured under standard conditions. The culture was terminated when the cell confluence in the control groups reached 80–90% (typically after approximately 10 days). The cells were then washed with Phosphate-Buffered Saline (PBS) and stained with crystal violet, and the visible colonies were counted^10^.

### Cell migration and motility assay

The wound healing assay was performed as previously described in our earlier study^10^ to evaluate the migratory capacity of breast cancer and non-tumorigenic breast epithelial cells following exposure to volatile anesthetic agents. For this assay, cells were seeded into six-well plates at a density of 1.5 × 10^5^ cells/well and grown to form a confluent monolayer. Following treatment with gas mixtures containing 3.6% sevoflurane or 10.3% desflurane (as previously described), a straight scratch was carefully created in the monolayer using a sterile 20-µl pipette tip.

Cellular debris was removed by washing with fresh medium, which was then replaced. Images of the scratched area were captured using light microscopy at 0 h, 24 h, and 48 h. Cell migration was quantified by measuring the distance traveled by cells at the leading edge of the wound. Results were expressed as the average distance between the edges of the gap at the specified time points^10^.

### Apoptotic cell death analysis

Apoptotic cell death was quantified by Annexin V–FITC/7-AAD staining followed by flow cytometric analysis as described above^10^. For this assay, MDA-MB-231, MCF-7, and MCF-10 A cells were seeded into 96-well plates (1 × 10^4^ cells/well) and treated with gas mixtures containing 3.6% sevoflurane or 10.3% desflurane (as described in the Volatile Agent Application section).

Following the treatment and incubation, cells were harvested and stained using the BioLegend FITC Annexin V Apoptosis Detection Kit with 7-AAD (Cat#640922) according to the manufacturer’s instructions. Flow cytometric analysis using a FACSARIA III flow cytometer (Becton Dickinson)^10^.

### Western Blot analysis

For Western blot analysis, MCF-7 and MDA-MB-231 cells (3.5 × 10⁵) were seeded in T-25 culture flasks and treated with 3.6% sevoflurane or 10.3% desflurane as described in the cell culture and volatile agent application section. Cells were collected, washed twice with ice-cold phosphate-buffered saline (PBS), and lysed in lysis buffer at 4 °C. Protein concentrations were measured using a DC protein assay kit (Bio-Rad, Hercules, CA, USA).

A total of 40 µg of protein from each sample was separated by SDS–polyacrylamide gel electrophoresis (4–20% gradient) and transferred to polyvinylidene difluoride membranes. Membranes were blocked in blocking buffer (0.1% Triton X-100 with 5% dry milk in Tris-buffered saline–Tween 20 (TBS-T)) for 60 min. After being washed with TBS-T, membranes were incubated with primary antibodies against PARP (Cell Signaling, #9542S), AIF (Cell Signaling, #5318S), p53 (Proteintech, 10442-1-AP), and β-actin (Proteintech, #60008-1-Ig).

After washing, membranes were incubated with horseradish peroxidase-conjugated secondary antibodies (Bio-Rad). β-actin was used as a loading control. Proteins were visualized using Clarity Western ECL Substrate (Bio-Rad) and imaged with a ChemiDoc MP Imaging System (Bio-Rad). Densitometric analysis was performed using Image Lab software (Bio-Rad).

### Statistical analysis

All experiments were performed in at least three independent biological replicates (*n* = 3). Data are presented as mean ± standard deviation (SD). Comparisons among multiple groups were performed using one-way analysis of variance (ANOVA) followed by Sidak’s multiple comparisons test. Normality of the data was assessed using the Shapiro–Wilk test, and homogeneity of variance was evaluated using Brown–Forsythe and Bartlett’s tests prior to analysis. Statistical analyses were performed using GraphPad Prism (version 10). A *p* value < 0.05 was considered statistically significant.

## Results

### Effect of desflurane and sevoflurane on the viability and proliferation of MDA-MB-231, MCF-7 breast cancer cells, and MCF-10 A non-tumorigenic breast epithelial cells

**Fig. 1:**
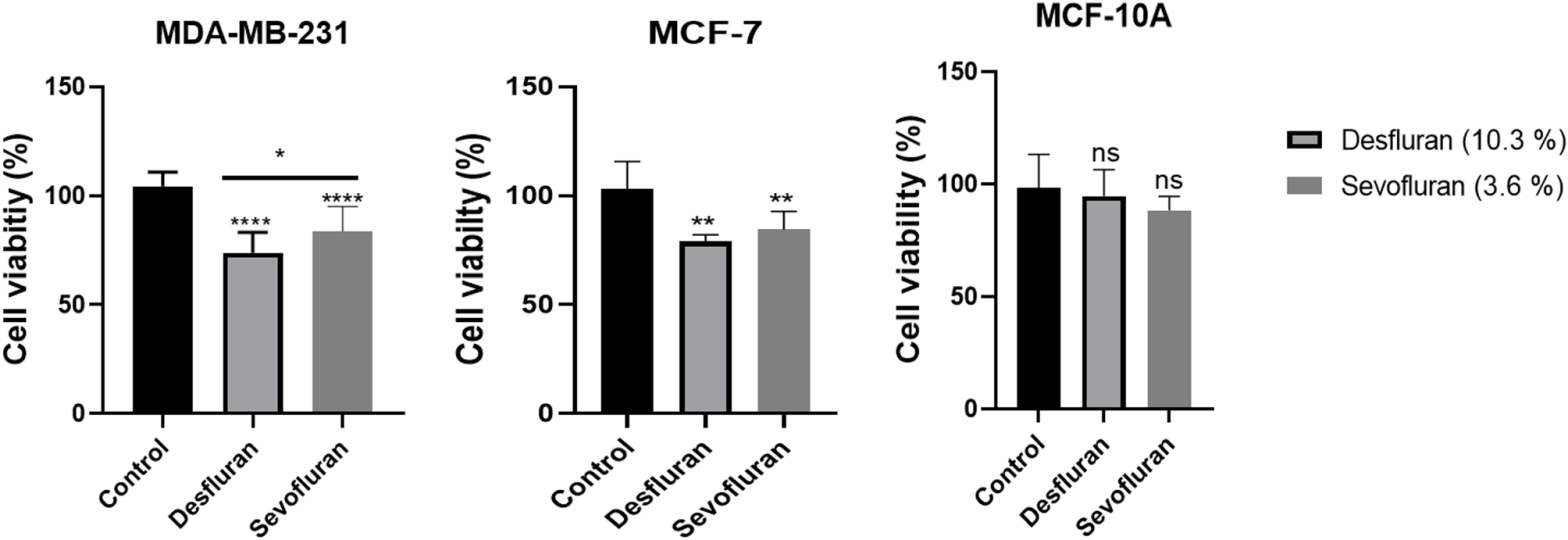
Effect of Desflurane and Sevoflurane on the viability of breast cancer cells (MDA-MB-231, MCF-7) and non-tumorigenic breast epithelial cells (MCF-10 A). Following 3 h of exposure and 24 h of subsequent incubation, both Desflurane and Sevoflurane significantly suppressed proliferation in cancer cells, while exhibiting no significant effect on normal epithelial cells. (****p < 0.0001, **p < 0.001, ns: non-significant). Data are presented as mean ± standard deviation (SD) from three independent biological replicates (*n* = 3). Statistical significance was determined using one-way ANOVA followed by Sidak’s multiple comparisons test.

Following 3 h of exposure to sevoflurane (3.6%) or desflurane (10.3%) and 24 h of subsequent incubation, a statistically significant reduction in cell viability was observed in both MDA-MB-231 and MCF-7 cells compared to the control group (Fig. 1).

In MDA-MB-231 cells, one-way ANOVA revealed a significant difference among the groups (F(2,35) = 43.09, *p* < 0.0001). Post hoc analysis demonstrated that both desflurane and sevoflurane significantly reduced cell viability compared to control (*p* < 0.0001 for both). Moreover, desflurane showed a significantly greater reduction in cell viability compared to sevoflurane (*p* = 0.0418).

In MCF-7 cells, one-way ANOVA also revealed a significant difference among the groups (F(2,20) = 13.30, *p* = 0.0002). Both desflurane and sevoflurane significantly reduced cell viability compared to control (*p* = 0.0006 and *p* = 0.0019, respectively), whereas no statistically significant difference was observed between the two anesthetic agents (*p* = 0.7223).

Conversely, no significant difference in cell viability was observed in MCF-10 A cells following exposure to either sevoflurane or desflurane compared to the control group (Fig. 1).

These findings indicate a subtype-specific response, with a more pronounced effect of desflurane observed in MDA-MB-231 cells.

### Effect of desflurane and sevoflurane on colony formation in MDA-MB-231, MCF-7 breast cancer cells, and MCF-10 A non-tumorigenic breast epithelial cells

**Fig. 2:**
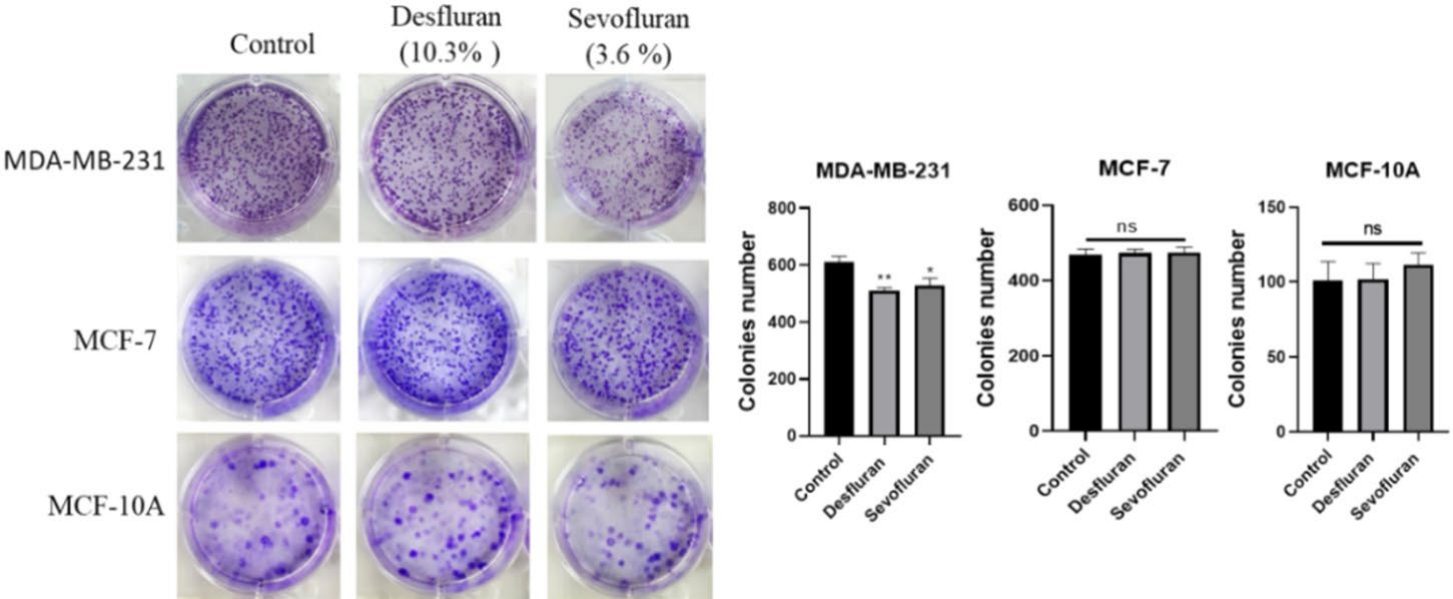
Effect of Desflurane and Sevoflurane on colony formation in breast cancer cells (MDA-MB-231, MCF-7) and non-tumorigenic breast epithelial cells (MCF-10 A). Following 3 h of exposure, colony formation was significantly suppressed in MDA-MB-231 cells, whereas no significant change was observed in MCF-7 and MCF-10 A cells. (**p < 0.01, *p < 0.05, ns: non-significant). Data are presented as mean ± standard deviation (SD) from three independent biological replicates (*n* = 3). Statistical significance was determined using one-way ANOVA followed by Sidak’s multiple comparisons test.

Clonogenic assays demonstrated a significant reduction in colony formation in MDA-MB-231 cells following exposure to desflurane (10.3%) or sevoflurane (3.6%) compared to the control group (Fig. 2). One-way ANOVA revealed a significant difference among the groups (F(2,6) = 23.64, *p* = 0.0014). Post hoc analysis showed that both desflurane and sevoflurane significantly suppressed colony formation compared to control (*p* = 0.0020 and *p* = 0.0056, respectively), whereas no statistically significant difference was observed between the two agents (*p* = 0.6381).

In contrast, no significant differences in colony formation were observed in MCF-7 and MCF-10 A cells following exposure to either anesthetic agent (Fig. 2).

### Effect of desflurane and sevoflurane on the migration of MDA-MB-231, MCF-7 breast cancer cells, and MCF-10 A non-tumorigenic breast epithelial cells

**Fig. 3:**
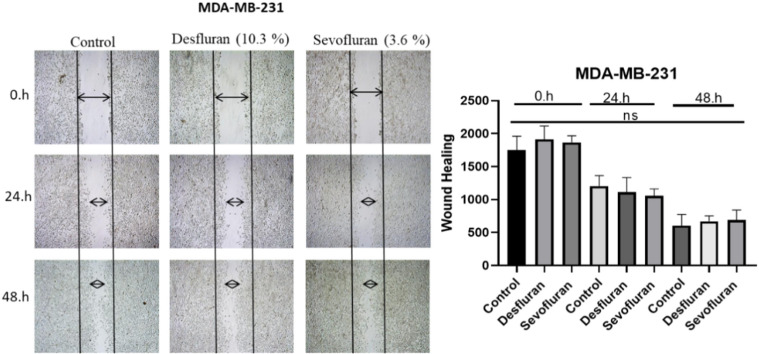
Effect of Desflurane and Sevoflurane on the migration of MDA-MB-231 breast cancer cells. Following 3 h of exposure, no significant change was observed in the migration of MDA-MB-231 cells compared to the control group in either the 24-hour or 48-hour evaluations (ns: non-significant). Data are presented as mean ± standard deviation (SD) from three independent biological replicates (*n* = 3). Statistical significance was determined using one-way ANOVA followed by Sidak’s multiple comparisons test.

**Fig. 4:**
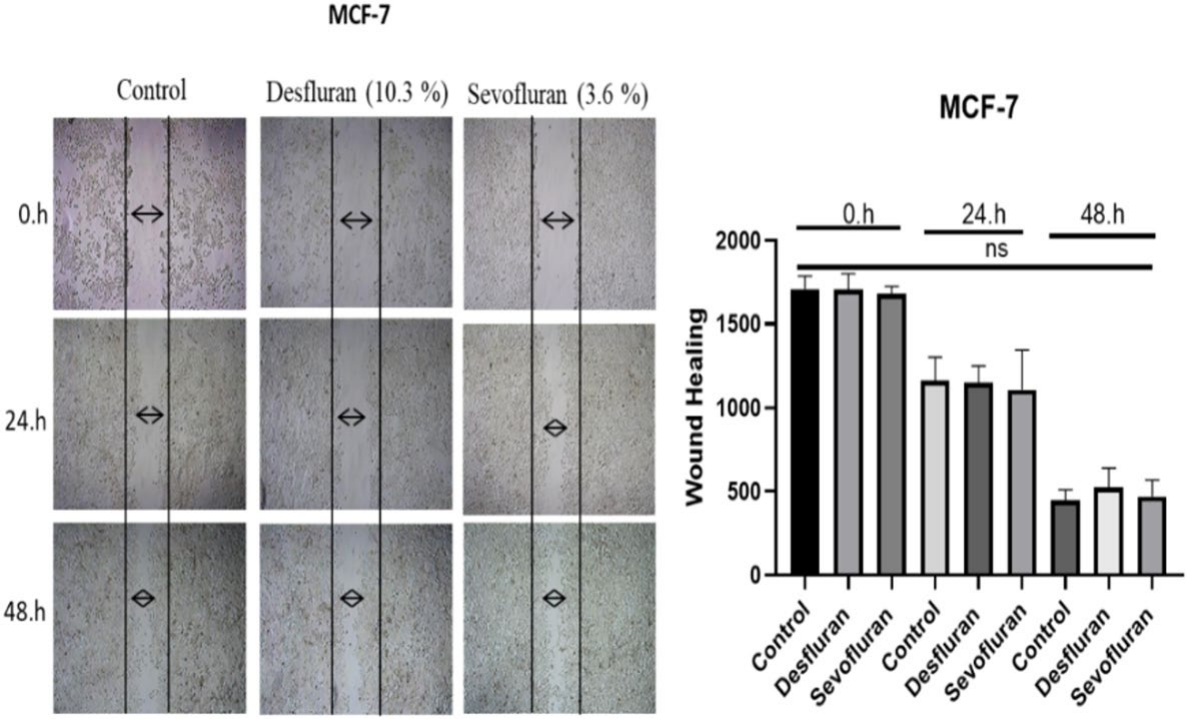
Effect of Desflurane and Sevoflurane on the migration of MCF-7 breast cancer cells. Following 3 h of exposure, no significant change was observed in the migration of MCF-7 cells compared to the control group in either the 24-hour or 48-hour evaluations (ns: non-significant). Data are presented as mean ± standard deviation (SD) from three independent biological replicates (*n* = 3). Statistical significance was determined using one-way ANOVA followed by Sidak’s multiple comparisons test.

**Fig. 5:**
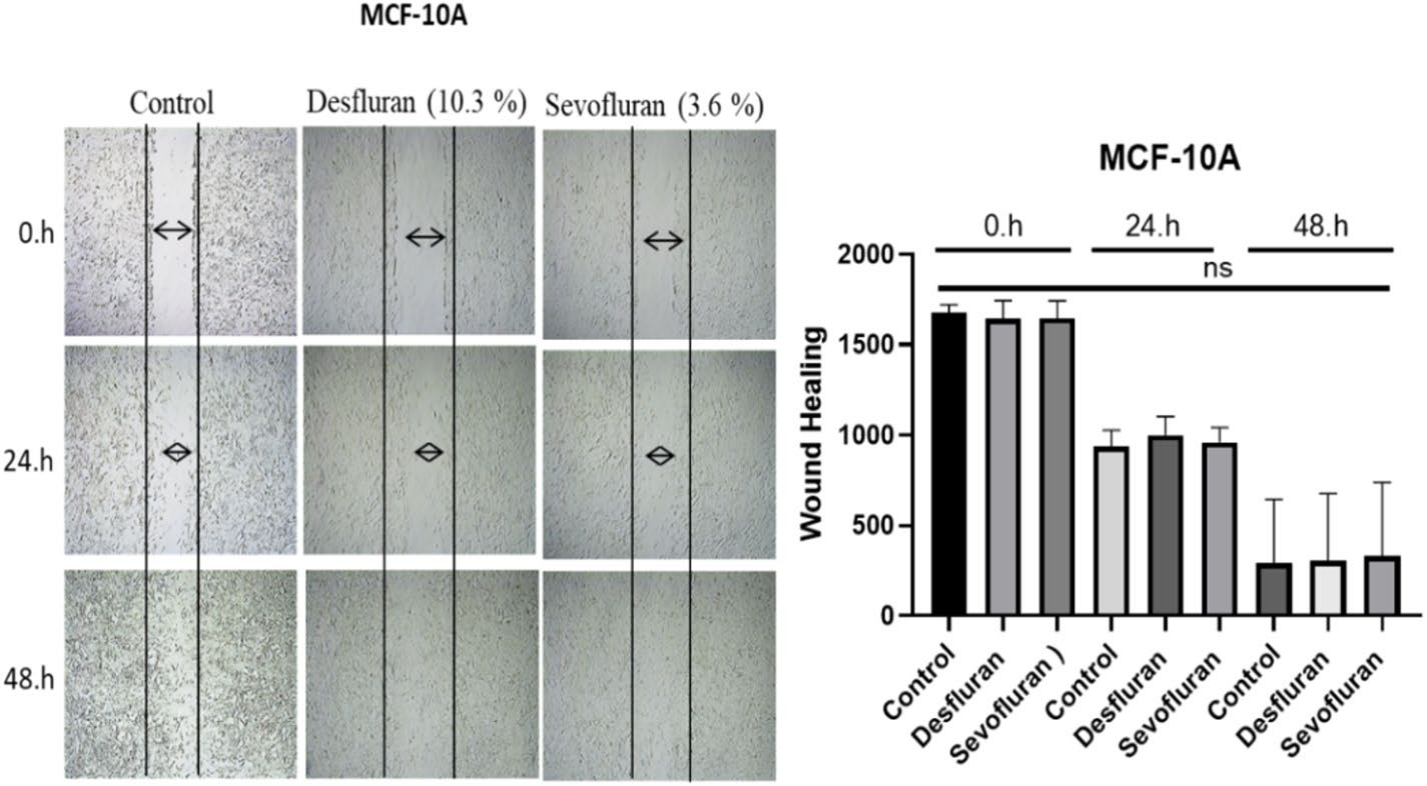
Effect of Desflurane and Sevoflurane on the migration of MCF-10 A non-tumorigenic breast epithelial cells. Following 3 h of exposure, no significant change was observed in the migration of MCF-10 A cells compared to the control group in either the 24-hour or 48-hour evaluations (ns: non-significant). Data are presented as mean ± standard deviation (SD) from three independent biological replicates (*n* = 3). Statistical significance was determined using one-way ANOVA followed by Sidak’s multiple comparisons test.

Wound healing assays revealed no statistically significant differences in migratory capacity among control, sevoflurane-treated, and desflurane-treated groups in MDA-MB-231, MCF-7, or MCF-10 A cells at 24–48 h (*p* > 0.05) (Figs. 3, 4 and 5). It should be noted that wound healing assays can reflect both cell migration and proliferation^28,29^. Therefore, the observed wound closure may not solely represent migratory activity. In addition, wound closure at 48 h may reflect the combined effects of both migration and proliferation. Therefore, the migration results should be interpreted with caution.

### Effect of desflurane and sevoflurane on apoptosis in MDA-MB-231, MCF-7 breast cancer cells, and MCF-10 A non-tumorigenic breast epithelial cells

**Fig. 6:**
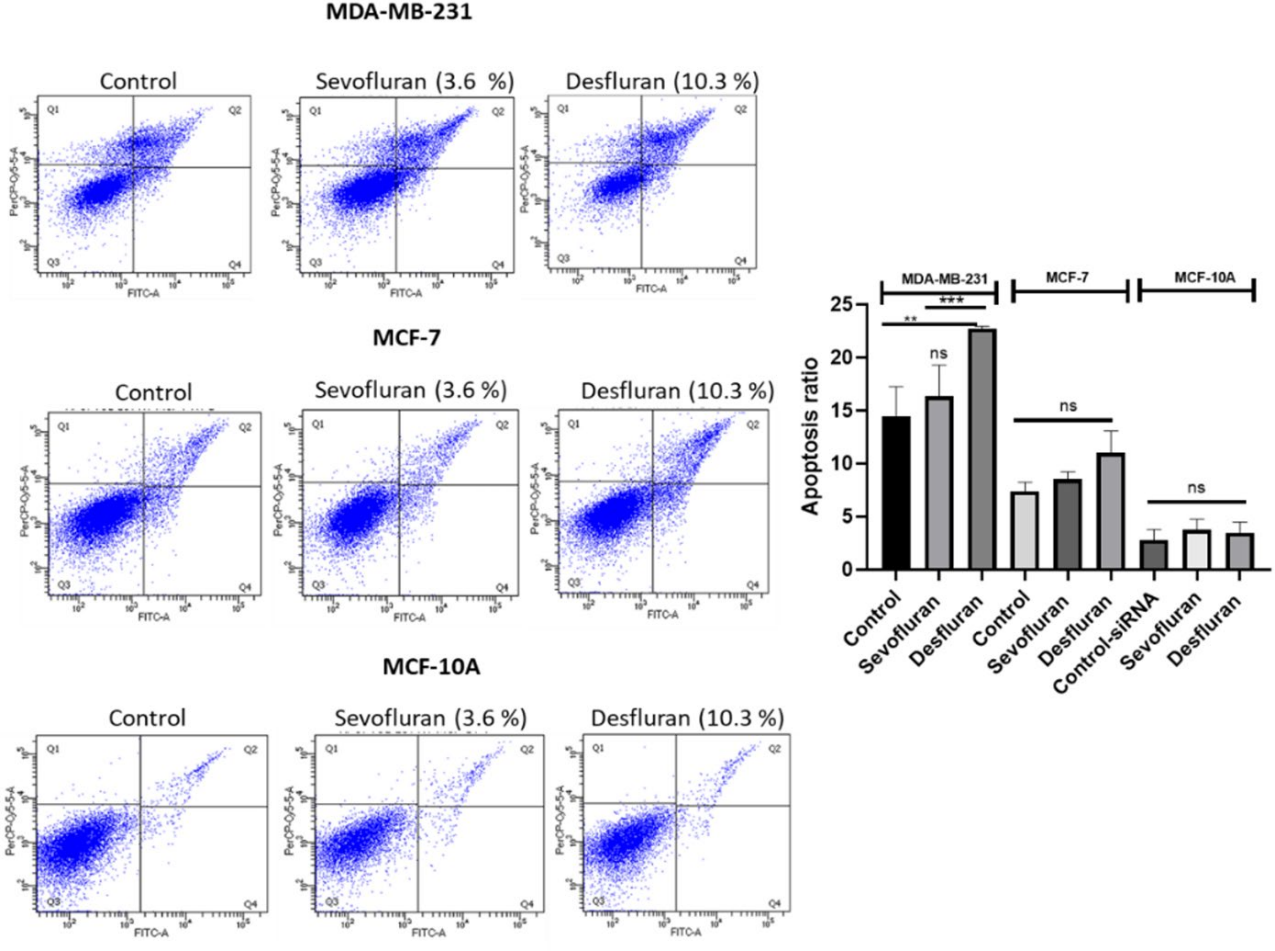
Effect of Desflurane and Sevoflurane on the rate of apoptosis in breast cancer cells (MDA-MB-231, MCF-7) and non-tumorigenic breast epithelial cells (MCF-10 A). Following 3 h of Desflurane exposure, the rate of apoptosis was significantly increased in MDA-MB-231 cells (*p* < 0.0001), while no significant change was observed after Sevoflurane exposure. Neither agent affected the rate of apoptosis in MCF-7 and MCF-10 A cells (ns: non-significant). Data are presented as mean ± standard deviation (SD) from three independent biological replicates (*n* = 3). Statistical significance was determined using one-way ANOVA followed by Sidak’s multiple comparisons test.

Annexin V–FITC/7-AAD flow cytometric analysis demonstrated a significant increase in apoptotic cell death in MDA-MB-231 cells following desflurane exposure compared to the control group (Fig. 6). One-way ANOVA revealed a significant difference among the groups (F(8,17) = 46.85, *p* < 0.0001). Post hoc analysis showed that desflurane significantly increased apoptosis compared to control (*p* < 0.0001), whereas sevoflurane did not result in a significant change (*p* = 0.4689). In addition, desflurane induced significantly higher levels of apoptosis compared to sevoflurane (*p* = 0.0008).

In contrast, no significant differences in apoptosis were observed in MCF-7 and MCF-10 A cells following exposure to either desflurane or sevoflurane compared to the control group (Fig. 6).

### Western blot analysis

**Fig. 7:**
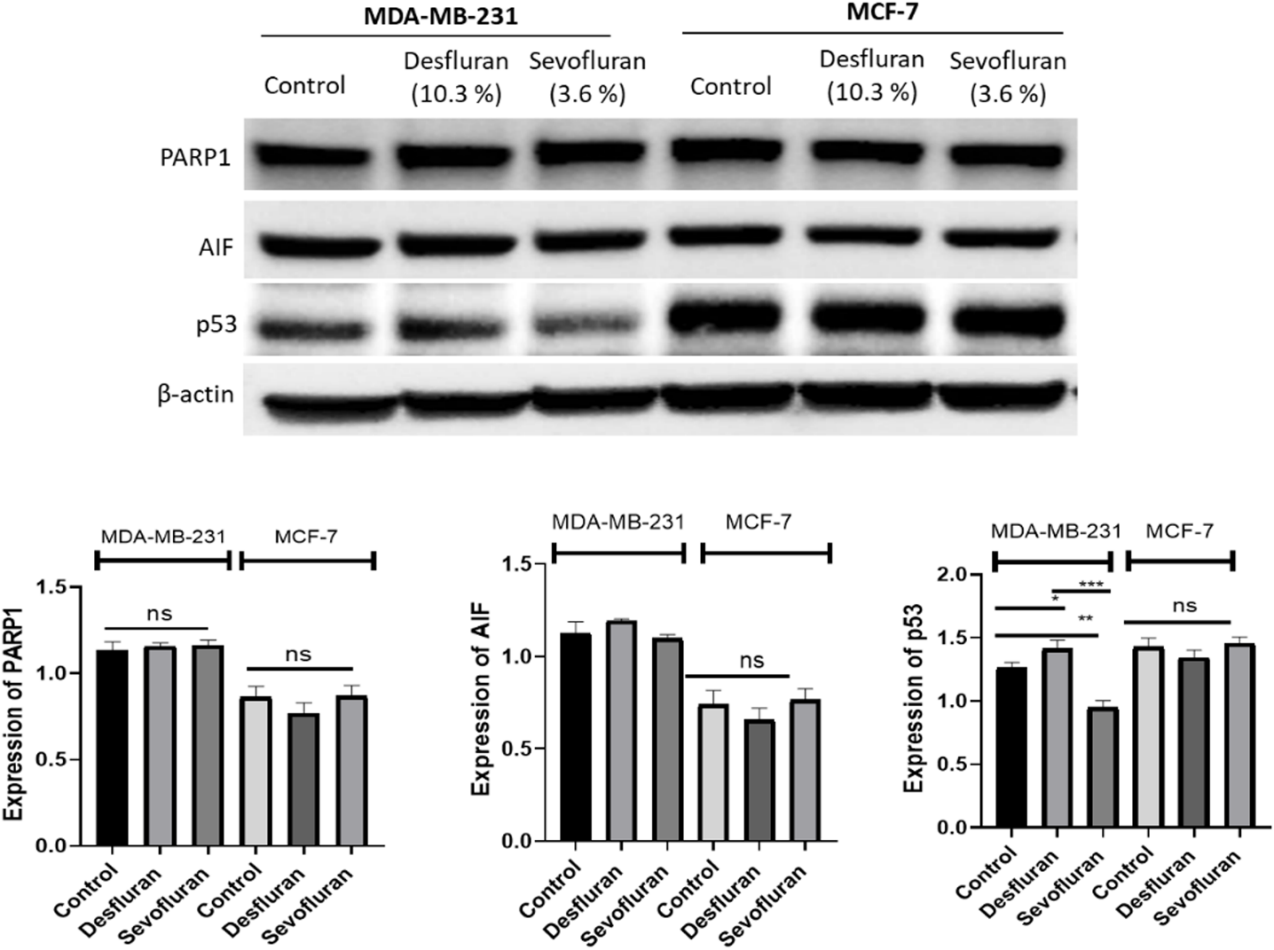
Effect of Desflurane and Sevoflurane on the expression of apoptosis-related proteins (PARP-1, AIF, and p53) in breast cancer cells (MDA-MB-231, MCF-7). Following 3 h of exposure in MDA-MB-231 cells, no change was observed in PARP-1 and AIF expression, while Desflurane increased p53 expression, and Sevoflurane decreased it. Neither agent affected the expression levels of PARP-1, AIF, and p53 in MCF-7 cells. (*p < 0.05, **p < 0.01, ns: non-significant). Protein expression levels were normalized to β-actin and quantified by densitometric analysis. Data are presented as mean ± standard deviation (SD) from three independent biological replicates (*n* = 3). Statistical significance was determined using one-way ANOVA followed by Sidak’s multiple comparisons test. Full-length uncropped blots corresponding to this figure are provided in Supplementary Figure S1.

Expression levels of proteins involved in the apoptotic pathway were evaluated by Western blot analysis following 3 h of exposure to desflurane (10.3%) and sevoflurane (3.6%) (Fig. 7).

In MDA-MB-231 cells, Western blot analysis revealed significant differences in p53 expression among the groups (one-way ANOVA, F (2,6) = 55.89, *p* = 0.0001) (Fig. 7). Post hoc analysis demonstrated that desflurane significantly increased p53 protein expression compared to control (*p* = 0.0475), whereas sevoflurane significantly reduced p53 expression (*p* = 0.0012). In addition, p53 levels were significantly higher in the desflurane-treated group compared to the sevoflurane-treated group (*p* = 0.0001).

No significant alterations were observed in PARP-1 or AIF expression following treatment with either anesthetic agent. In MCF-7 cells, the expression levels of PARP-1, AIF, and p53 remained unchanged among control, desflurane-treated, and sevoflurane-treated groups (Fig. 7).

## Discussion

In the present study, we comparatively investigated the direct biological effects of desflurane and sevoflurane on breast cancer cell lines representing different molecular subtypes under in vitro conditions. Our findings indicate that desflurane more effectively suppressed proliferation and colony formation and induced apoptotic cell death in triple-negative MDA-MB-231 cells, accompanied by increased p53 expression. In contrast, sevoflurane showed relatively weaker antiproliferative effects and was associated with reduced p53 levels.

Interestingly, neither anesthetic agent significantly affected proliferation or apoptosis in MCF-7 or MCF-10 A cells, and no significant differences in wound closure were observed in any of the investigated cell lines. However, it is important to consider that wound healing assays reflect not only cell migration but also cell proliferation^28,29^. In our experimental setting, where proliferation was significantly reduced, the contribution of cell division to wound closure may still play a role. As such, the observed wound closure may not exclusively represent migratory activity. Moreover, at later time points such as 48 h, cell proliferation is likely to contribute to wound closure, further limiting the ability to attribute these results solely to migration.

The influence of anesthetic technique on cancer recurrence and long-term oncological outcomes remains controversial. While several retrospective clinical studies have compared total intravenous anesthesia (TIVA) and inhalational anesthesia in breast cancer surgery, the reported associations with survival and recurrence have been inconsistent^12–14^. For example, Yoo et al.^14^ reported no significant difference in mortality between TIVA and inhalational anesthesia in a large retrospective cohort of breast cancer patients. However, retrospective design, lack of molecular subtype stratification, and absence of agent-specific subgroup analyses limit the mechanistic interpretation of these findings. In this context, preclinical studies provide important insights into the direct cellular effects of anesthetic agents independent of systemic immune modulation.

Previous in vitro investigations examining the effects of sevoflurane on breast cancer cells have yielded conflicting results. Hirai et al.^15^ reported increased proliferation in several cancer cell lines, including MDA-MB-231, whereas proliferation was suppressed in MCF-7 cells. Deng et al.^16^ demonstrated enhanced viability mediated through modulation of intracellular calcium homeostasis, while Tiron et al.^17^ showed that sevoflurane promoted epithelial–mesenchymal transition via AKT isoform regulation. Conversely, Liu et al.^18^, Zeng et al.^19^, and Wu et al.^20^ reported antiproliferative and anti-invasive effects mediated through microRNA-dependent pathways. Ecimovic et al.^3^ observed increased proliferation and migration following sevoflurane exposure. These discrepancies likely reflect differences in exposure duration, concentration, molecular subtype, and experimental conditions. In our experimental model, short-term exposure to clinically relevant concentrations of sevoflurane reduced proliferation in MDA-MB-231 cells without significantly altering apoptosis, suggesting that growth inhibition may occur through mechanisms distinct from classical apoptotic pathways.

Compared with sevoflurane, fewer studies have directly examined the effects of desflurane on breast cancer cells. Woo et al.^21^ reported preservation of immune cell populations following desflurane anesthesia in breast cancer patients, suggesting a potentially less immunosuppressive profile. In colorectal cancer models, desflurane has been associated with both reduced viability at higher concentrations^22^ and enhanced metastatic behavior through microRNA-mediated pathways^23^. Furthermore, Zhao et al.^24^ demonstrated that volatile anesthetics may differentially modulate proliferation and migration depending on tumor type. In ovarian cancer cells, desflurane exposure has been shown to influence metastasis-related gene expression^7,25^, while contrasting effects were observed in neuroglioma models^26^. Liu et al.^27^ reported no significant differences among inhalational agents in an in vivo murine breast cancer model. Collectively, these findings highlight the tumor-type and context-dependent nature of volatile anesthetic effects. In contrast to previous studies reporting variable and sometimes conflicting effects of volatile anesthetics, the present study provides a systematic and direct comparison of desflurane and sevoflurane across distinct breast cancer subtypes under standardized experimental conditions, offering novel insight into their subtype-specific biological effects.

In the present study, desflurane-induced upregulation of p53 expression in MDA-MB-231 cells was observed in parallel with increased apoptosis. As a central regulator of cell cycle arrest and programmed cell death, p53 is classically associated with tumor suppressive pathways. However, given that MDA-MB-231 cells harbor mutant p53, the observed increase in p53 expression may reflect stabilization and accumulation of non-functional protein rather than activation of canonical tumor suppressor mechanisms. Under standard conditions, increased expression of mutant p53 (mutp53) in these cells is associated with enhanced malignancy^30,31^. However, our results demonstrate that desflurane-induced upregulation of p53 correlates with increased apoptosis and reduced viability. This apparent discrepancy suggests that desflurane may engage alternative or p53-independent pro-apoptotic signaling pathways, or potentially modulate the functional state of mutant p53 under stress conditions.

The absence of significant alterations in PARP-1 and AIF expression suggests that desflurane-induced apoptosis may involve selective activation of specific signaling cascades rather than broad mitochondrial dysfunction. Importantly, the lack of significant effects in MCF-7 and MCF-10 A cells supports the notion that volatile anesthetics may exert subtype-specific biological effects, particularly in aggressive triple-negative breast cancer cells^2,3^.

It is important to note that the present findings are based on short-term in vitro exposure and do not directly reflect long-term clinical outcomes such as tumor recurrence or patient survival. While clinical studies provide insight into patient outcomes, in vitro studies offer mechanistic understanding at the cellular level; therefore, these results should be interpreted within the context of experimental conditions.

Several limitations should be acknowledged. This study was conducted under in vitro conditions and therefore does not capture the complexity of interactions within the tumor microenvironment, immune system, or systemic physiological responses. As a result, the findings may not fully reflect tumor behavior in vivo, and further validation in in vivo models and clinical settings is needed to better understand their translational relevance. In addition, only short-term exposure effects were evaluated, and downstream molecular signaling pathways were not explored in detail.

Volatile anesthetics may exert concentration- and time-dependent effects that are influenced by cellular context; however, the present study was not designed to establish a formal dose–response relationship. Future studies exploring a broader range of concentrations and exposure durations would help to better characterize their potential dose-dependent biological effects. Collectively, these findings provide a basis for future work aimed at understanding the potential role of anesthetic agents in cancer biology.

Taken together, our findings demonstrate that desflurane exerts a more pronounced antiproliferative and pro-apoptotic effect compared to sevoflurane in triple-negative breast cancer cells under controlled in vitro conditions. These subtype-specific differences highlight the importance of considering tumor biology when interpreting the potential effects of anesthetic agents. However, further in vivo and clinical studies are required to determine whether these cellular effects translate into meaningful differences in oncological outcomes.

## Supplementary Information

Below is the link to the electronic supplementary material.

Supplementary Material 1

## Data Availability

The datasets generated during and/or analysed during the current study are available from the corresponding author on reasonable request.
