# Supplementary material for: Research on the effects of desflurane and sevoflurane on proliferation and migration of breast cancer cells
Source: Sci Rep. 2026 May 9;16:21300. doi: 10.1038/s41598-026-52017-5 (PMC13346580; doi:10.1038/s41598-026-52017-5)
Supplement: Supplementary file 1 — Supplementary Material 1 [file 41598_2026_52017_MOESM1_ESM.pdf]

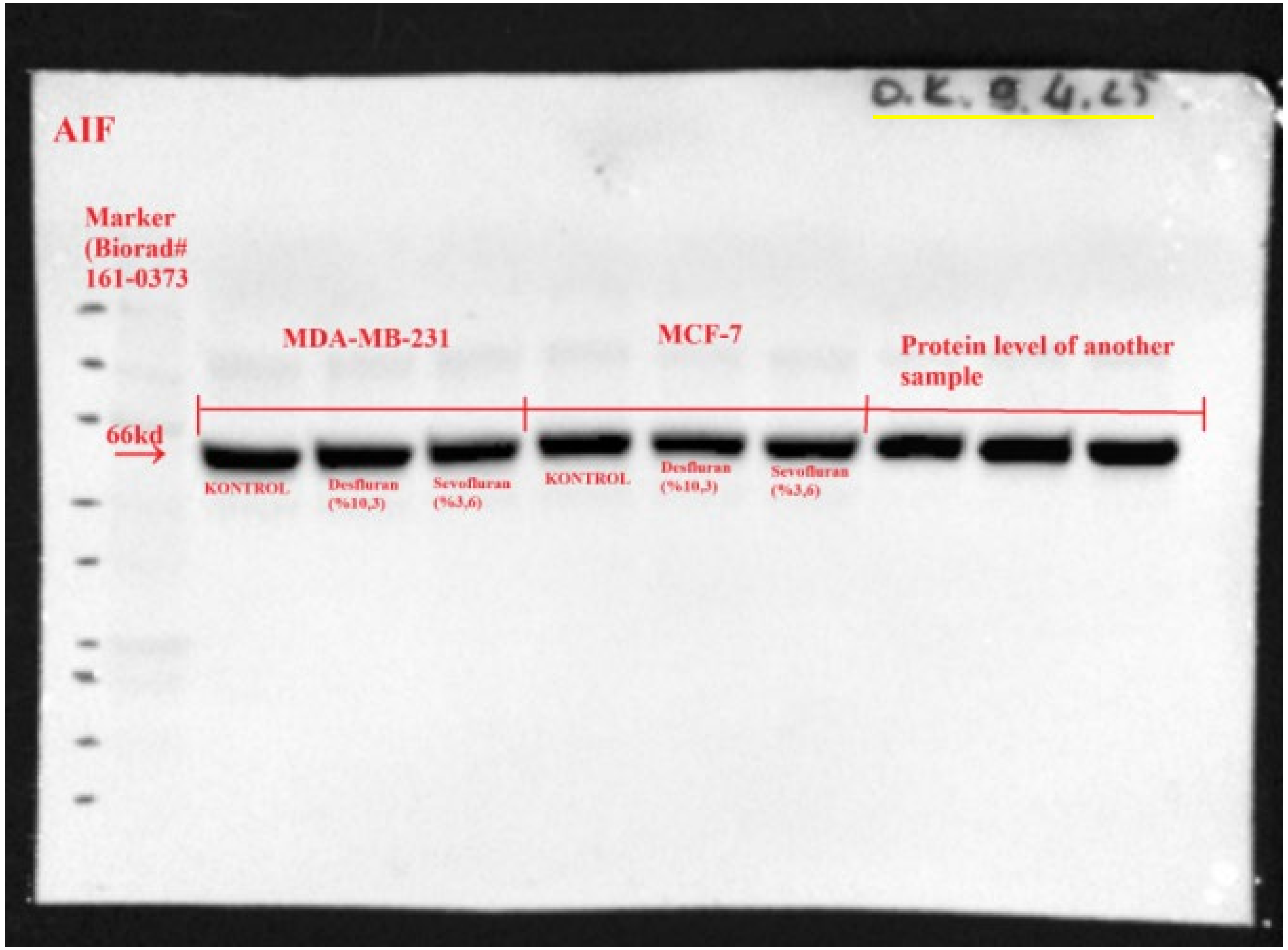

D.K. 9.4.25
AIF
Marker (Biorad# 161-0373
66kd
MDA-MB-231
MCF-7
Protein level of another sample
KONTROL
Desfluran (%10,3)
Sevofluran (%3,6)
KONTROL
Desfluran (%10,3)
Sevofluran (%3,6)

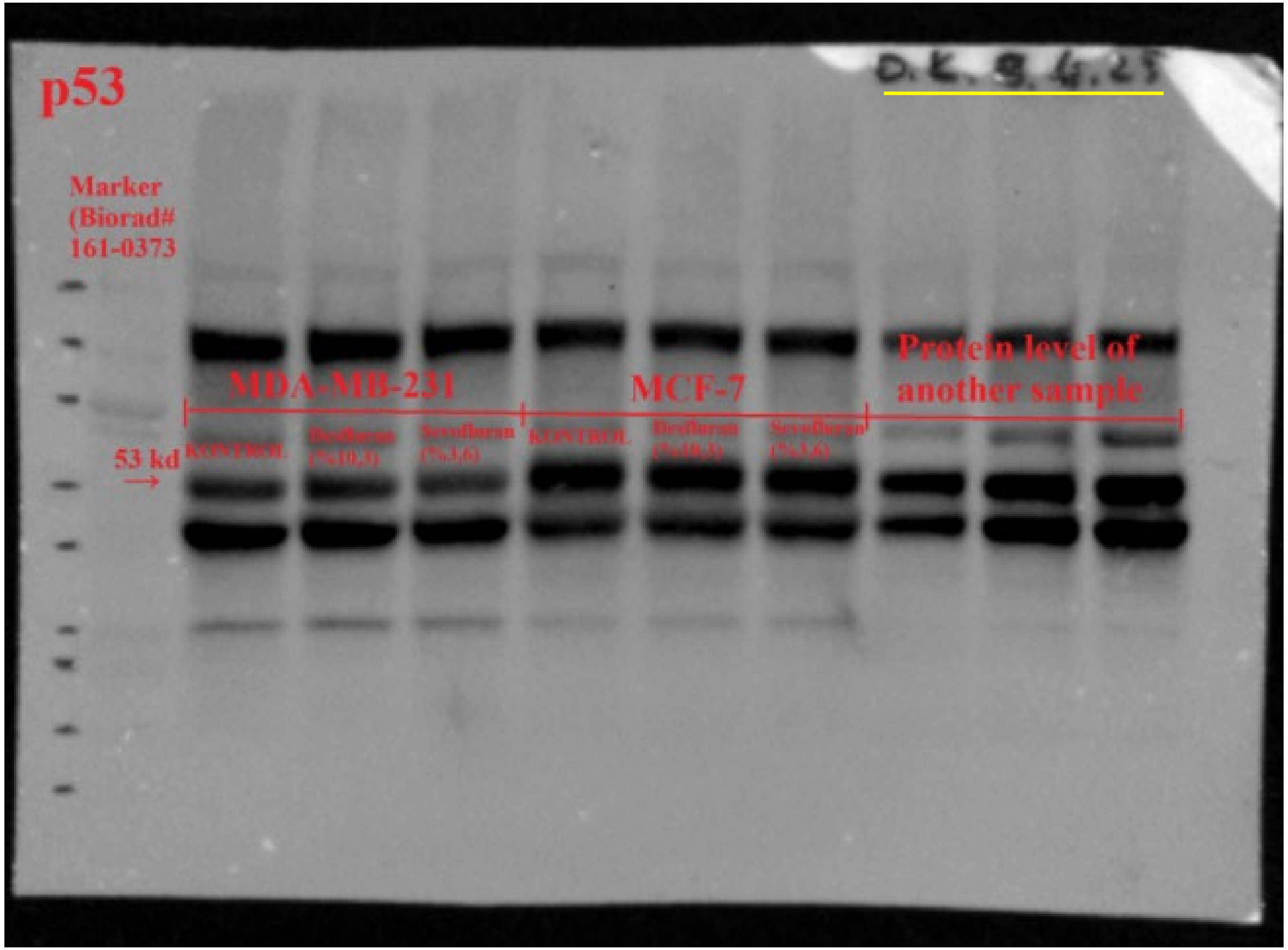
p53
Marker
(Biorad#
161-0373
53 kd
MDA-MB-231
MCF-7
Protein level of
another sample
KONTROL
Desfluran
(%10,3)
Sevofluran
(%3,6)
KONTROL
Desfluran
(%10,3)
Sevofluran
(%3,6)

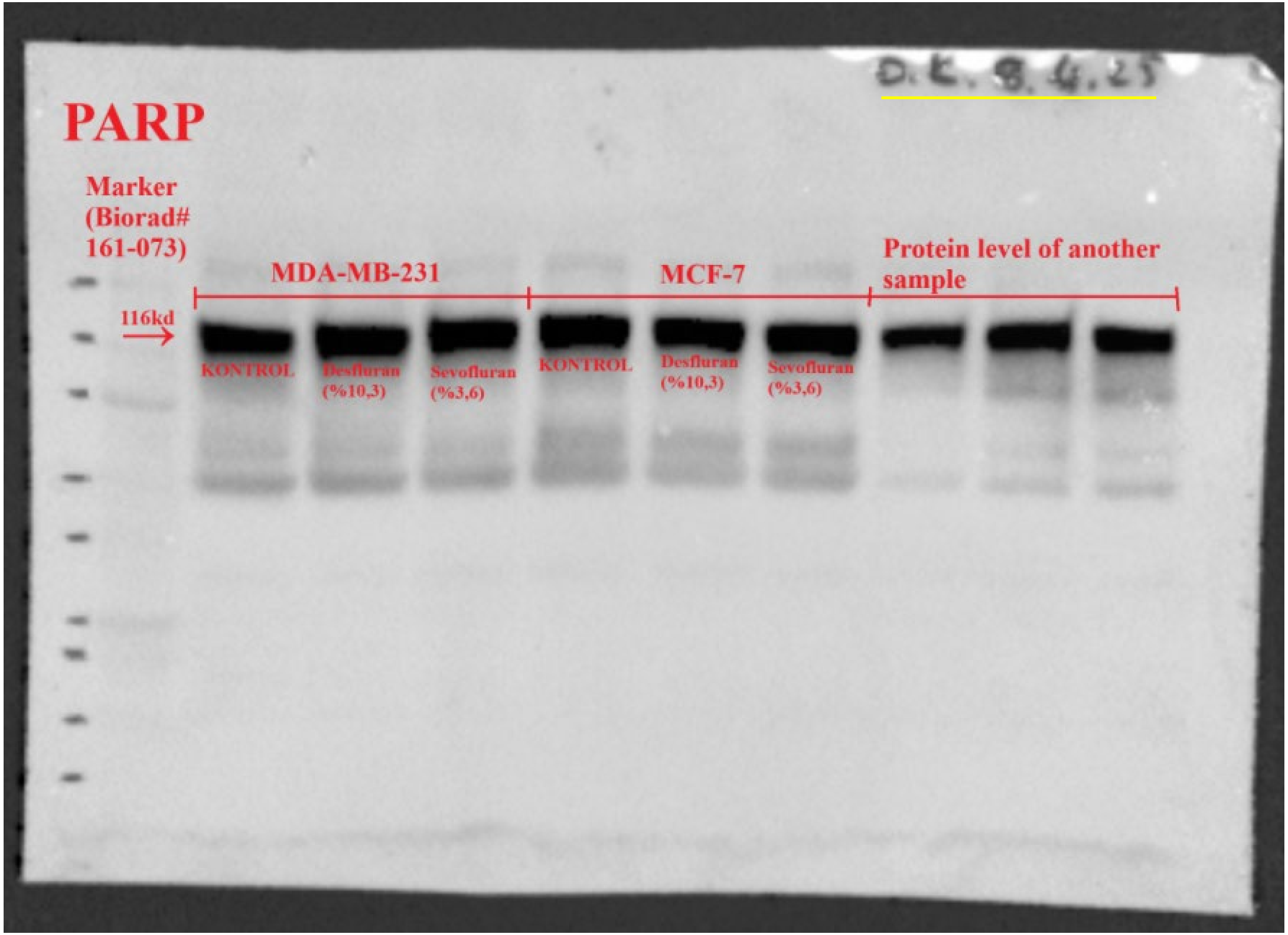
PARP
D.K. 8.4.25
Marker (Biorad# 161-073)
116kd
MDA-MB-231
MCF-7
Protein level of another sample
KONTROL
Desfluran (%10,3)
Sevofluran (%3,6)
KONTROL
Desfluran (%10,3)
Sevofluran (%3,6)

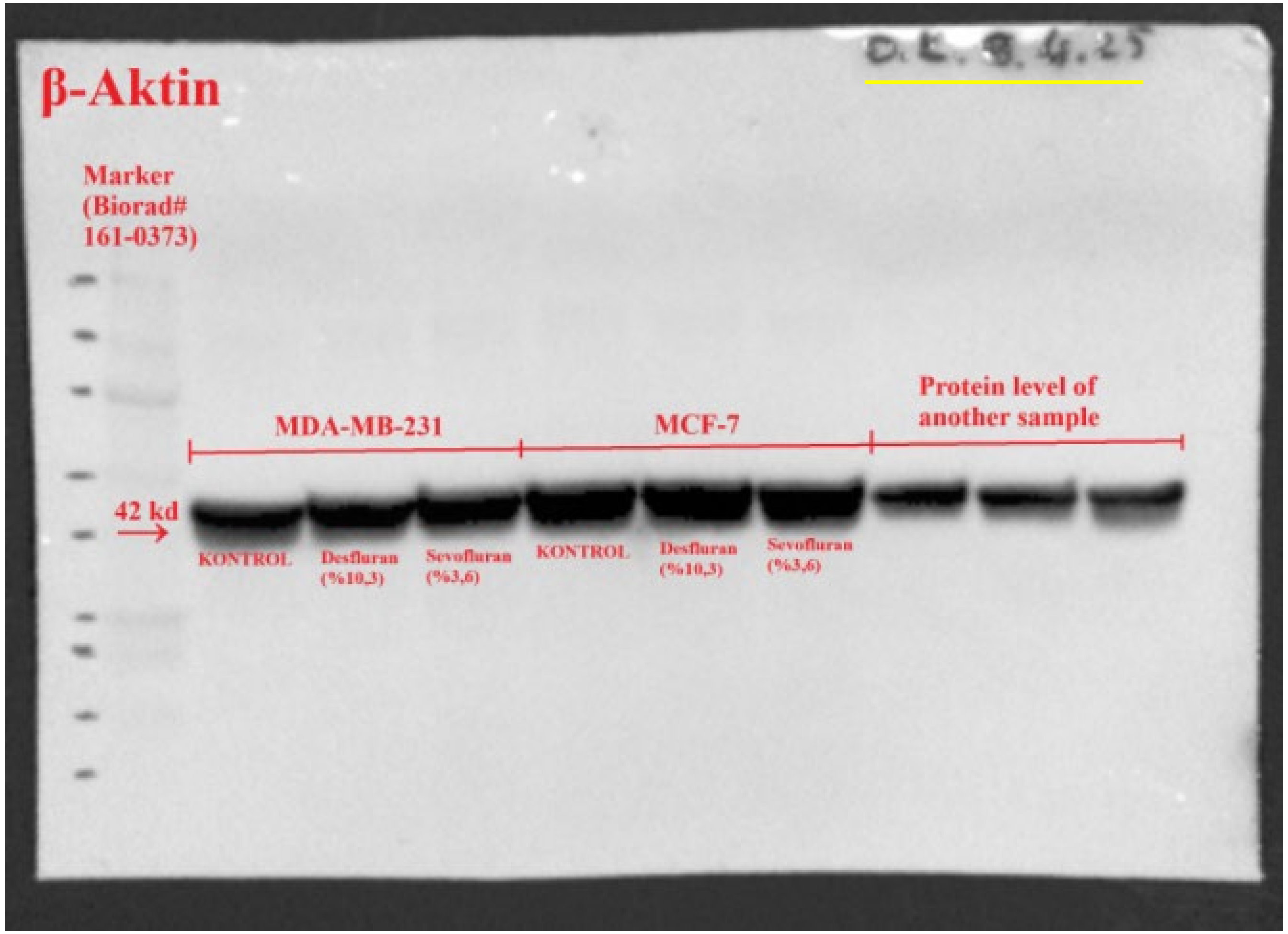

Supplementary Figure (S1)
